# The Global Fund’s paradigm of oversight, monitoring, and results in Mozambique

**DOI:** 10.1186/s12992-017-0308-7

**Published:** 2017-12-12

**Authors:** Ashley Warren, Roberto Cordon, Michaela Told, Don de Savigny, Ilona Kickbusch, Marcel Tanner

**Affiliations:** 1 Department of Epidemiology and Public Health, Swiss Tropical and Public Health Institute, Socinstrasse 57, 4002 Basel, Switzerland; 2 University of Basel, Petersplatz 1, 4003 Basel, Switzerland; 3 Franklin University Switzerland, Via Ponte Tresa 29, 6924 Lugano-Sorengo, Switzerland; 4 Graduate Institute of International and Development Studies, Maison de la Paix, Chemin Eugène-Rigot 2, 1202 Geneva, Switzerland

**Keywords:** Global Fund, Mozambique, Financial management, Performance-based finance, Coordination, Country oversight, Reform, New funding model

## Abstract

**Background:**

The Global Fund is one of the largest actors in global health. In 2015 the Global Fund was credited with disbursing close to 10 % of all development assistance for health. In 2011 it began a reform process in response to internal reviews following allegations of recipients’ misuse of funds. Reforms have focused on grant application processes thus far while the core structures and paradigm have remained intact. We report results of discussions with key stakeholders on the Global Fund, its paradigm of oversight, monitoring, and results in Mozambique.

**Methods:**

We conducted 38 semi-structured in-depth interviews in Maputo, Mozambique and members of the Global Fund Board and Secretariat in Switzerland. In-country stakeholders were representatives from Global Fund country structures (eg. Principle Recipient), the Ministry of Health, health or development attachés bilateral and multilateral agencies, consultants, and the NGO coordinating body. Thematic coding revealed concerns about the combination of weak country oversight with stringent and cumbersome requirements for monitoring and evaluation linked to performance-based financing.

**Results:**

Analysis revealed that despite the changes associated with the New Funding Model, respondents in both Maputo and Geneva firmly believe challenges remain in Global Fund’s structure and paradigm. The lack of a country office has many negative downstream effects including reliance on in-country partners and ineffective coordination. Due to weak managerial and absorptive capacity, more oversight is required than is afforded by country team visits. In-country partners provide much needed support for Global Fund recipients, but roles, responsibilities, and accountability must be clearly defined for a successful long-term partnership. Furthermore, decision-makers in Geneva recognize in-country coordination as vital to successful implementation, and partners welcome increased Global Fund engagement.

**Conclusions:**

To date, there are no institutional requirements for formalized coordination, and the Global Fund has no consistent representation in Mozambique’s in-country coordination groups. The Global Fund should adapt grant implementation and monitoring procedures to the specific local realities that would be illuminated by more formalized coordination.

**Electronic supplementary material:**

The online version of this article (10.1186/s12992-017-0308-7) contains supplementary material, which is available to authorized users.

## Resumo

O Fundo Global é uma das ONGs mais importantes do mundo quando se trata do desenvolvimento da saúde global. Em 2015 foi desembolsado quase dez por cento de todo o orçamento à assistência no mundo direcionada à saúde. Em 2011, iniciaram um processo de reforma em resposta a alegações do mau uso das doações recebidas. As reformas têm focado no processo de aplicação do dinheiro, já as estruturas da ONG e seu paradigma tem permanecido intactos.

Esta análise trata os resultados das discussões sobre o Fundo Global, seu paradigma de supervisão e monitoramento, e os resultados derivados em Moçambique. Pesquisadores realizaram 37 entrevistas em pessoa e uma entrevista por telefone com 38 partes interessadas em Maputo, Moçambique e membros do Conselho e Secretariado do Fundo Global na Suíça. Os entervistados em Moçambique eram representantes das estruturas locais do Fundo Global **(**p. ex. destinatários principais), do Ministério da Saúde, e ‘agencias bilaterais ou multilaterais e adidos da saúde ou desenvolvimento de embaixadas parceiras em Moçambique. Foram também incluídos representantes das Nações Unidas, consultores, e coordenadores das ONGs.

Codificação temática revelou preocupações quanto à combinação da supervisão fraca no país com os requisitos rigorosos de monitoramento e avaliação; processos que estão conectados ao financiamento baseado em desempenho. A análise revelou que, apesar das mudanças associadas ao *New Funding Model (Novo Modelo de Financiamento)*, a maioria dos consultados, tanto em Genebra como em Maputo, reconhecem que aínda existem desafios na estrutura e no paradigma do Fundo Global. A falta de um escritório em Moçambique tem alguns efeitos negativos, causando dependência em parceiros nacionais e também ineficácia na coordenação.

Devido à fraca capacidade de absorção e de gerenciamento no pais, é necessária mais supervisão da que é possível atualmente nas visitas do gerente de carteira em Genebra. Os parceiros em Moçambique fornecem apoio aos beneficiários do Fundo, mas as diferentes tarefas, responsabilidades e prestações de contas precisam ser definidas mais claramente para conseguir uma parceria bem-sucedida a longo prazo. Além disso, os tomadores de decisão em Genebra reconhecem que a coordenação local é imperativa para uma implementação bem-sucedida; os parceiros continuam a apreciar o aumento do envolvimento do Fundo em Moçambique. Atualmente, não existem requisitos institucionais para uma coordenação formal, e o Fundo não tem uma presença continua dentro de grupos de coordenação em Moçambique. O Fundo Global precisa moldar a implementação de projetos e procedimentos de monitoramento às realidades locais que seriam destacadas através de uma coordenação formalizada.

## Background

The Global Fund to Fight AIDS, Tuberculosis, and Malaria (Global Fund) is a financial instrument established in early 2002 [1]. Its formation was part of the “emergency response to accelerate the scale-up of control of the major communicable diseases, especially HIV/AIDS” in light of the Millennium Development Goals (MDGs) [2]. Governments provide approximately 95% of Global Fund support; the private sector provides the rest [3]. Since its inception, it has disbursed more than US$30.6 billion [4]. In 2015, the Global Fund was the world’s largest channel of finance for malaria and tuberculosis (40% and 49% of total support, respectively), and the second largest channel for HIV/AIDS (16% of total support). In terms of overall contribution, the Global Fund was responsible for 9 % of funding for global health in 2015; it reached its maximum in 2012 and 2013 when it oversaw the disbursement of 12% of the total funds dedicated to development assistance for health [5, 6].

The Global Fund has seven core structures, the: Board, Office of the Inspector General, Technical Review Panel, Principle Recipient, Country Coordinating Mechanism, Staff / Secretariat, and Local Fund Agent [7]. The Global Fund Board is the overall governing body responsible for defining policies, objectives, and strategies. It includes representatives from both donor and recipient countries, non-governmental organizations (NGOs), the private sector, and affected communities [8]. The Office of the Inspector General (OIG) is an independent body that oversees investment effectiveness including risks associated with misused funds. The Technical Review Panel (TRP) is an independent team of health and development experts that evaluates proposals submitted to the Global Fund [9].

The Principle Recipient (PR) is responsible for grant implementation and can either be part of the public sector, e.g. a ministry, an NGO, or even a private company. The PR is under the direct supervision of the Country Coordinating Mechanism (CCM). The CCM is reflective of the Global Fund’s dedication to local ownership and decision-making. It writes the original grant proposal, nominates implementers, and governs grant implementation. The CCM is a partnership of country stakeholders including the private sector, academic institutions, multilateral and bilateral development partners, civil society, and key affected populations [9].

The Global Fund Secretariat is responsible for the daily operations, primarily grant management. The Secretariat engages with Principal Recipients through country teams. The Global Fund does not have offices in recipient countries. Instead it uses Local Fund Agents (LFAs) to oversee grant management [9].

In October 2010 the Global Fund received its largest replenishment, to date, at US$11.7 billion despite allegations of illicit use of funds in Zambia, the Philippines, and Mauritania and subsequent freezing of their cash disbursements [10–13]. At the 22nd Board Meeting, the OIG released its Progress Report for March through October 2010. It outlined its findings from investigations of allegations of fraud, corruption, and misuse of funds in seven countries [14]. On 23 January 2011, the Associated Press published a story, “Fraud Plagues Global Health Fund” [15]. More than 250 media outlets worldwide covered the story and within days Germany froze its contribution to the Global Fund [16]. Over the coming months the Global Fund underwent independent review, re-visited its 5-year strategy, and committed to urgent reform [17–19]. Within one year, its Executive Director, Michel Kazatchkine, announced that he would step down, cutting his tenure two years short [17, 20]. Gabriel Jaramillo, former Chairman and Chief Executive Officer of Sovereign Bank, was appointed as General Manager to oversee the transformation plan [21]. Mr. Jaramillo lacked technical expertise in health development, but he specialized in managing change in complex financial institutions during his 36 years of experience in banking in Latin and South America and the US [22].

The OIG systematically audited recipients and identified US$118 million in losses as of 19 September 2013 [23]. It is important to note that these losses are only 0.5% of the US$22.7 billion that the Global Fund had disbursed worldwide at the time [24]. Overall, the Global Fund has a particularly high level of financial accountability, compared with other global health agencies, and is diligent in its response to these relatively small abuses.

### The new funding model

Reforms resulted in what became known as the New Funding Model (NFM). It has five key characteristics: flexible timeline, simplified grant application processes, shorter approval processes, enhanced engagement of all partners prior to grant submission, and improved predictability of funding [25]. In short, the reform focuses on processes, not structure or paradigm.

Initially the Global Fund application process was in distinct rounds announced by a call for proposals approximately three months before a submission deadline. In the NFM, funding cycles are flexible and countries can submit a so-called concept note any time during windows. This allows countries to align the grant timeline with national fiscal years and strategies. Countries are eligible to apply for a pre-assigned amount per disease, called “the envelope”. Envelopes are determined by countries’ burden of disease and ability to finance. This approach is meant to enhance predictability of funding [25].

**Fig. 1:**
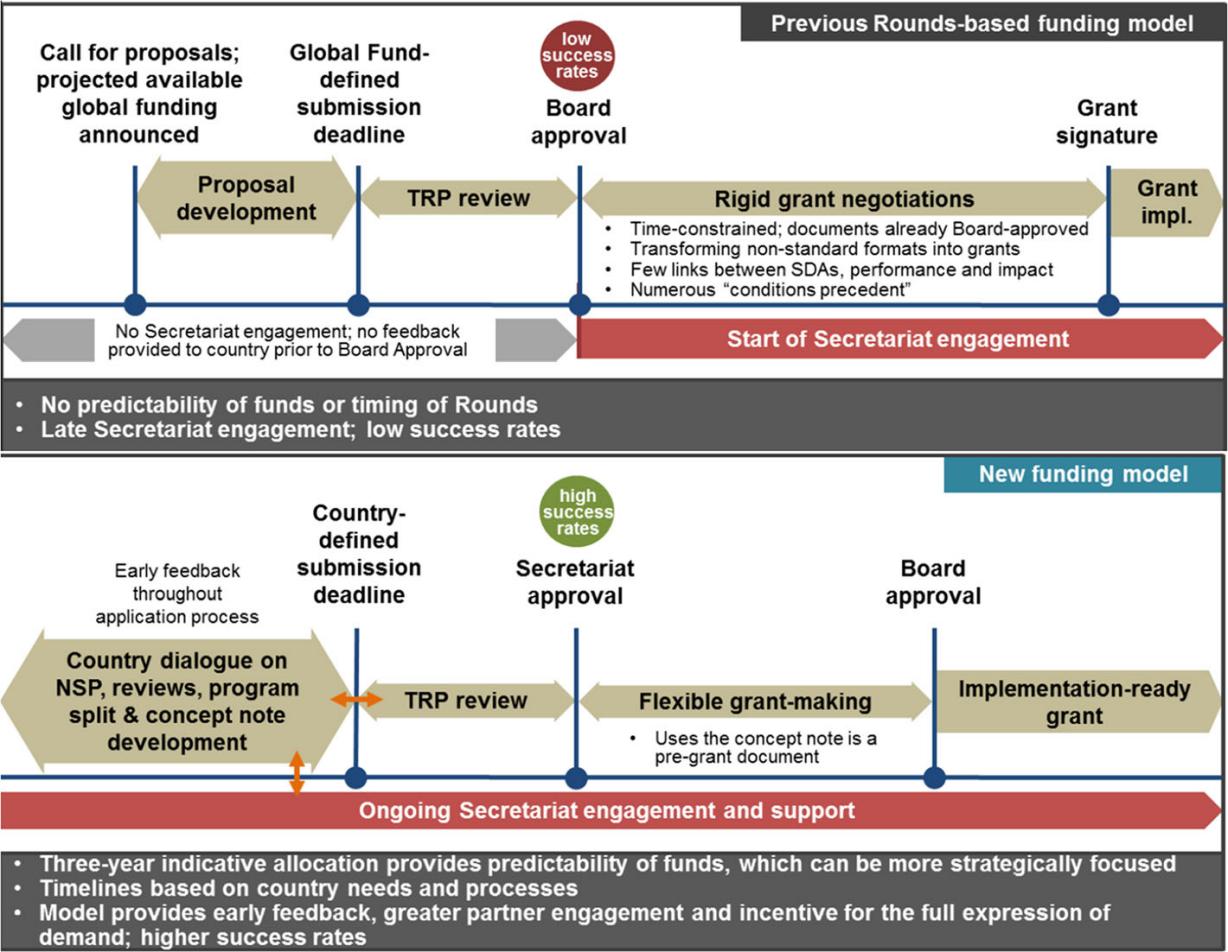
Comparison of the Rounds-based funding process and the New Funding Model [26]

CCMs seek technical assistance to write grants for the three diseases (and health systems strengthening (HSS) which can either stand alone or be incorporated into a disease-oriented grant). Upon submission, grants are screened for eligibility by the Secretariat and then passed along to the TRP which recommends technically sound proposals for funding. The Board gives official approval of chosen grants. The grants undergo classifications and budget cuts by the Board before being returned to the TRP for negotiations, further reductions of the budgets through efficiency gains, and division between multiple PRs (and the subsequent necessary modifications to the budget). Then PRs and the Global Fund sign the final grant agreement The most notable change in the NFM is that from the beginning, country teams are engaged in country-level dialog on concept note development (Fig. 1).

### The Global Fund in Mozambique

As of 2016, Mozambique has been awarded 17 grants. The Global Fund signed, committed, and disbursed a total of more than US$972 million, US$802 million, and US$620 million, respectively, to Mozambique in its fight against the three diseases. (Please note that the discrepancy between values of signed, committed, and disbursed is due to active grants.) The average portfolio is US$466 million making Mozambique the 12th highest recipient of Global Fund support [3].

Of Mozambique’s 17 grants, 12 have been awarded to the Ministry of Health, representing 86% of funds disbursed to Mozambique. From 2004 and 2008 the Global Fund disbursed US$135.8 m into a health financing basket, known as PROSAUDE [27]. PROSAUDE was part of the state budget and was the common fund for development assistance in health (the basket fund) of the Mozambique sector-wide approach to health sector funding.

In early grants, scorecards issued at the end of Phase I often reported “weak financial management practices and capabilities within the MOH resulting in weak financial accountability for resources used” along with difficulties tracking funds in PROSAUDE [28–32]. In 2011, in response to calls for increased accountability, the OIG began an audit of Global Fund grants to Mozambique’s Ministry of Health for years 2008, 2009, and 2010 [27]. Five months into the audit they concluded a total of US$3,318,395 was inadequately accounted for. The OIG recommended that the Ministry of Health repay the PROSAUDE. Overall, they concluded that there were not “adequate controls … in place to manage the key risks impacting the Global Fund supported programs” [27].

This study was conducted to understand how the Global Fund was experienced by key stakeholders in Maputo, Mozambique and how recent reforms were experienced by key stakeholders in Mozambique as well as by Global Fund stakeholders in Geneva, Switzerland. In late 2013 members of the research team conducted interviews with 38 representatives from these two groups.

## Methods

### Primary data collection

Data was collected as part of a larger study on the influence of emerging donors in health development [33]. For the purpose of the larger study “emerging donors” includes public-private partnerships, philanthropic foundations, corporate social responsibility programmes, Brazil, Russia, India, China, South Africa (the BRICS) [34], and other emerging economies.^1^ The investigators conducted 37 face-to-face and one phone-based semi-structured in-depth interviews with stakeholders in Maputo, Mozambique and members of the Global Fund Board and Secretariat in Switzerland. In-country stakeholders were representatives from Global Fund country structures (e.g. Principle Recipient), the Ministry of Health, health or development attachés of partner embassies in-country, selected UN agencies, consultants, and the NGO coordinating body (Table 1). Interviews were held in the offices of key informants in Switzerland and Maputo, Mozambique. Interviews lasted approximately one hour but ranged from 45 min to three hours.

**Table 1:** Interviewees by representation

Representation	Number of interviewees	Nomenclature in paper
Global Fund Board	5	GFBoard^b^
Global Fund Secretariat	5	GFSecretariat^b^
Global Fund Country Structure^a^	4	GFCountryStructure^b^
Academia	1	Academia^b^
Consultant Firm	2	Consultant^b^
Non-governmental organization	3	NGO^b^
Multilateral Agency	6	Multilateral^b^
OECD Partner	10	OECDPartner^b^
Coordination Body	2	Coordination^b^
Total	38	

Location of interviews: 11 of the GF and 4 of the OECD and Multilateral Agency interviews were conducted in Geneva. The remaining 23 interviews were conducted in Mozambique

^a^includes Local Fund Agents, Country Coordinating Mechanism, Principal Recipients, and Sub-Recipients; members of the CCM may be a representative of a multilateral agency or OECD Partner (Organization for Economic Cooperation and Development)

^b^refers to interviewees in chronological order

Investigators contacted prospective interviewees via email. We obtained email addresses through professional contacts and official websites. Emails contained a brief description of the research team, overall research questions and objectives, and methods. Respondents suggested additional interviewee(s) who were then contacted directly by the research team.

Interviewers used a semi-structured interview guide (Additional file 1). Discussions included questions about interviewees’ perspectives on overall changes in development assistance for health and resulting influences at country-level. Interviewees were also asked to share their opinions about who they perceived to be the most influential private sources of finance including the Global Fund. Many of interviewers’ questions were in the context of the Global Fund as an international non-governmental organization as opposed to an intergovernmental organization or a purely bilateral donor. We asked open-ended questions about Global Fund engagement with the government and other donors. Interviewers allowed respondents to lead discussion for the most part and followed up with more detailed questions for clarification. Interviewers also asked questions based on insights provided in earlier interviews.

The 15 interviews conducted in Switzerland followed a similar structure to those in Mozambique. Five of these interviews were conducted much later, in mid-2015, after an initial content analysis, and focused on the Global Fund and the New Funding Model, specifically (Academic1, NGO1, GFCountryStructure1, GFBoard4 and 5, and GFSecretariat5).

In Mozambique 23 interviews were conducted. The discussions were primarily in English with periodic clarifications in Portuguese as one investigator speaks Portuguese fluently. In Switzerland, 11 interviews were conducted with 13 interviewees. All discussions were in English. One phone-based interview was conducted in English, and a second member of the in-country NGO coordination body provided input via email in response to later follow-up.

Key informants in Mozambique received follow-up questions nine months after the initial interview. Of the 24 interviewees, eleven responded and two referred the investigators to new respondents who provided insights.

### Analysis

The corresponding author combined interviewers’ notes into one Microsoft Word document per interviewee and organized the material into fundamental themes- type of donor, aid management, health system, country context, etc. The corresponding author uploaded interview notes into MAXQDA 11 (UdoKuckartz; Berlin, Germany) and read each at least three times. Each successive reading was accompanied by descriptive, analytic, and thematic coding, respectively, to assemble discussion points on the Global Fund paradigm and country-level engagement. The interview notes were indexed using the framework and included sub-themes as determined by the initial analytic coding [35].

To maintain respondents’ anonymity, each interviewee was given a label with the following nomenclature: two letter country code, professional affiliation, and number (based on chronological order of interviews of people with same professional affiliation). For example, “OECDPartner2” for the second OECD Partner interviewed.

## Results

Each country in the larger four-country study on emerging donors for health had a different experience and relationship with the Global Fund, but the general tone of response in Mozambique was that of a question- does the current approach of the Global Fund fit with country-level needs? Most interviewees were active before, during, and after a protracted audit and were left with concerns about the future of the Global Fund in Mozambique as it undergoes its first phase of reform. *Global Fund wants to follow only its own rules, and here it is not working* (Multilateral3). Perhaps the Global Fund’s inflexibility to recipient needs is because it *tends to be obsessed with financial technicalities* (Multilateral2). Respondents in both Mozambique and Switzerland voiced concerns about the combination of weak country presence/oversight/guidance with stringent and cumbersome requirements for monitoring and evaluation (M&E) linked to performance-based financing (Table 2). They felt this combination forces buttressing by partners. Respondents in Geneva also expressed concern that perceived weaknesses of partners add risk in this paradigm.

**Table 2:** Summary of country-level perceptions of the Global Fund’s paradigm

Aspect of paradigm	Perceived country-level result	Respondent(s)
Performance-based financing	• Recipients’ focus on disbursement rather than results	OECDPartner10
	• Burdensome administrative requirements	GFCountryStructure4
	• Duplication of reporting efforts from the ground all the way to central level	Multilateral3
Emphasis on financial technicalities	• Staff with financial rather than development background who lack country experience	GFBoard4
Lack of country office	• Other partners doing monitoring for the Global Fund	OECDPartner2
	• Global Fund is not engaged in country-level coordination	Coordination1, OECDPartner3
	• Forces partners to coordinate among themselves more	Multilateral2
	• Frequent deadlines and time stress	GFSecretariat5
	• Over-worked staff, communication challenges, out-of-touch with realities on the ground	GFSecretariat5
	• Dependent on expertise and interest of single person (Fund Portfolio Manager)	GFBoard4, GFCountryStructure1, GF Secretariat5
Partnerships	• Reliance on external consultants to develop proposals	Multilateral2
	• Early identification of gaps and provision of additional support	OECDPartner6
	• Undefined roles and concerns about accountability	GFBoard2, GFCountryStructure4
	• Potential for agenda alignment with single partner and less coordinated/multilateral approach	GFBoard1, GFCountryStructure2, GFSecretariat5, OECDPartners2, 3, and 10

There appears to be a strong association in the minds of respondents in Mozambique between performance-based funding, financial tracking, and the audit. This results in concerns about the Global Fund’s overall approach. *There have been a lot of discussions about Global Fund’s inflexibility and unrealistic demands of a developing country. ‘You are asking for oranges, but we only have bananas’* (OECDPartner1). Some respondents were more hopeful that distance from the audit will change the relationship with the Global Fund. *The Global Fund has a horrendous story with performance-based financing. There have been improvements in the past two years* (OECDPartner2). Both of these quotes illustrate a disconnect between the perception of Performance-based finance (PBF) at headquarter-level and at the country-level. In Geneva they believe this approach will ultimately help countries identify obstacles to meeting national goals and incentivize problem-solving. In Mozambique this approach feels based on implausible expectations and a resulting frustration.

Interviewees in Mozambique and Geneva agreed there are problems associated with the current paradigm of country oversight. *The Global Fund has weak country presence. Much more oversight is required; they must do more than disburse funds* (OECDPartner10). *Something that is missing in the Global Fund’s current approach is close contact with the realities on the ground. There is also a high level of internal movement that affects continuity* (GFBoard4). GFBoard4 commented, *[e]mployees need a strong financial background rather than focusing on a background in development or field experience*. This was linked to the discussion about the Global Fund’s quickness to remind that “we are a funder, not a development agency.” This contributes to the perception the Multilateral2 voiced, *Global Fund tends to be obsessed with financial technicalities* **.**

The Global Fund reportedly uses effective coordination as a criterion for grant approval. *The success of the application for Phase II of Round 9’s grants for HIV/AIDS and malaria was contingent upon the coordination among partners. So, effective coordination among partners is recognized as a potential weakness in Mozambique* (Coordination1). However, as there is no country presence, the Global Fund itself doesn’t participate in coordination bodies. Engagement with the Health Partners Group would greatly enhance the Global Fund’s understanding of country-level activities as this coordination body is *a clearing house of what each partner is doing* (OECDPartner3).

The technical support provided by partners is an integral component of the principle recipients’ success and, therefore, the Global Fund’s success. *The amount of support buttressing the Global Fund’s activities depends on the country* (GFCountryStructure1). In Mozambique, *[w]e still have to rely on external consultants to develop our proposals* (Multilateral2). Discussions in Geneva revealed that there is tension among stakeholders regarding reliance on technical partners and how these relationships are financed. *On the one hand, if the Global Fund is not going to develop its own technical capacity, it has to be able to rely on useful, helpful, actionable guidance from the partners and I think that has been a real problem* (GFBoard2). Furthermore,

*The big problem has been basically from the creation of the Fund that technical partners are very important; they are the ones who are present on the ground … And many of the countries depend on the technical assistance and the guidance from these technical partners. … It works already quite well in some countries and less well in others. And the challenge is to get more consistent, let’s say, quality of technical assistance provided by these partners* (GFSecretariat1).

## Discussion

### History of the Global Fund in Mozambique

Mozambique first received Global Fund support in Round 2 (year 2004) for all three diseases. The Global Fund signed Mozambique’s first Memorandum of Understanding (MoU) for the sector-wide approach, the PROSAUDE basket fund. It channelled resources through the basket until the audit of Round 7. Due to problems with reporting and tracing expenditure, the Global Fund paused funding for Round 8 (year 2008) [36–39]. Meanwhile the second PROSAUDE MoU was released; the Global Fund did not sign.

The Round 8 grant expired while awaiting results of the OIG’s Round 7 audit. Continued support during the audit would have required a parallel system for operation. Interviewees gave different accounts of the audit. Those who work closely with the Global Fund described it very matter-of-factly. To paraphrase: It was initially claimed that US$14 million was unaccounted for, but after one year, the audit deemed US$1 million missing. This is not surprising given the nature of pooled funding. Eventually the government repaid the US$1 million (OECDPartner1). But one interviewee from a bilateral agency was more cutting in response,

*In standard auditing protocol, the organization would be given three months to provide proper evidence for spending. The Global Fund gave three months, three times. … This is not good practice. The Global Fund hid behind its procedures. They did not grant access to their draft reports even though they audited bilateral funding by nature of auditing pooled funding. This is not a healthy approach. They are not improving health or a health sector when covering up results this way* (OECDPartner10).

Three years after the audit, the Round 8 Health System Strengthening grant was re-constituted in June 2012. The Ministry of Health is the principle recipient, and the grant focuses on risk mitigation and reporting as per the OIG’s report. Despite the Global Fund reforms in application processes, *[the grant proposal] went through the old bureaucratic processes* (OECDPartner1).

In Mozambique the audit, requirements for financial management, and performance-based approach, have inspired questions about Global Fund’s paradigm given the contexts in which it works. One quote in particular encapsulates many of the concerns addressed piece-meal in other interviews.

*The Global Fund has two choices: either continue to not have people on the ground or relax their requirements for monitoring and evaluation. As it stands there is no one on the ground, they maintain their high expectations, and have other partners doing their monitoring for them* (OECDPartner2).

### The Global Fund paradigm

The Global Fund highly values recipient ownership in program development, implementation, and evaluation [40]. The PR leads grant application, administers funds, develops their targets for performance-based funding, and tracks results [41]. Due to this emphasis on country ownership, the Global Fund operates without country offices [42]. Communication with the Global Fund is entirely dependent on country teams. Often the PR seeks technical assistance, particularly for grant application and evaluating performance [43]. Additionally recipients are typically supported, to some degree, by development partners on the ground [44]. Bilateral agencies working in the country provide support, some more than others, because they also have vested interest in Global Fund’s success as they are donors to the Global Fund [45].

The Global Fund is a financier, rather than an implementing/development agency. Along with other global health initiatives created in the early 2000s, it was designed to overcome market and public failures in international public health, as well as disperse the power of the UN and its agencies [46–48]. It was meant to offer streamlined, less bureaucratic, processes. Respondents in Mozambique suggested that the Global Fund currently functions somewhere between its predecessors and the vision of its creators.

*The Global Fund has straddled between a managerial and a bureaucratic model in Mozambique. They try to apply performance-based financing, but their grant management processes have been highly bureaucratic. They function as a bureaucracy, but they’d like to have performance-based targets* (OECDPartner1).

The cost of the bureaucracy extends beyond cumbersome administrative processes … [s]*o much money spent on managing and getting through the bureaucratic requirements of the Global Fund* (OECDPartner1).

Despite respondents’ concerns, they were hopeful about prospective changes with the New Funding Model, and more importantly about the capacity of the Global Fund to reform at all. *The Global Fund’s New Funding Model addresses inefficiency concerns, and the recent changes in the Global Fund have shown how international organizations are capable of re-inventing themselves* (GFCountryStructure2).

The Global Fund’s reform is unique for a large-scale organization [49, 50], and respondents seemed to be providing constructive criticism with the hope that feedback would result in further reform of the Global Fund.

### Performance-based funding

Results-based financing is an attempt to link financial input to health-related outcomes. Development partners compare the results of the funded project or program to pre-determined targets for a set of indicators. Although this approach is not new, there is a broader range of actors using a wider range of results-based models. If implemented properly, results-based finance can: align donor and recipient objectives, improve data reliability, give recipients a stake in the outcome of their efforts, and give recipients greater discretion and authority to carry out their tasks [51]. On the other hand there are concerns about the effectiveness of these tools for health development [52] and the feasibility of measuring outcomes of complex, system-level interventions [53, 54]. To date, studies have focused on approaches that focus on paying for the results achieved by individuals or institutions (for example, health facilities or central medical stores). There has only been one study on results-based approaches to grant management [37].

Performance-based finance (PBF) is one of the guiding principles of the Global Fund; continued support for recipients depends on proven results. Their strategy to “actively manage grants based on impact, value for money and risk” includes increased emphasis on impact of funding, investment in data systems, requirement of increased financial management transparency, coordination with recipients and other donors, and avoidance of duplicated or inconsistent demands on recipients [55].

GFSecretariat2 shared that the organization uses a more progressive model of PBF than a strictly results-based approach. Currently the approach centers upon progress and improved performance of national programs. They focus on country ownership of results and corrective actions. The Global Fund wants to understand why a country is not performing, not just where the money is going. *Part of this decision-making is to get countries themselves to do their own performance reviews. So part of it is not just the mechanical rating, it’s that they actually do review their results against their targets, and they explain the deviations and they come to an overall rating* (GFSecretariat2).

While this approach is arguably more holistic, it has been reasoned that the subjective elements of their evaluation decrease incentives to improve performance [37]. This conclusion was supported by a development partner in Mozambique. *The recipients’ criterion for success is disbursement rather than results* (OECDPartner10).

The Global Fund approach was described as a streamlined skeleton which countries built upon to create their own performance evaluation. *…we had several hundred indicators and we’ve reduced it down to a top set of 10, which are highly weighted, but then a country can use further indicators. They have to set targets, and then it’s really how many of those targets are reached* (GFSecretariat2). But concerns at the country-level focus on burdens placed on data collection systems. *There is an enormous amount of paperwork to fill out … All data that is not aggregated in the routine national health information system must be gathered in the programme* (GFCountryStructure4). Multilateral3 shared that Mozambique has performed well and accomplished targets despite the obstacles posed by Global Fund requirements.

Mozambique does not necessarily have the financial management capacity required to satisfy Global Fund demands, and this directly affects return-on-investment measurements. Additionally, addressing the obstacles identified in the performance evaluation would require systems support.

*They give more money than such a weak system can properly absorb. So overall they might be doing more harm than good. Giving such a large sum of money without the proper checks and balances leads to corruption and growing inequality* (OECDPartner10).

Mozambique is not alone in this. Low absorption capacity has been blamed for the failure of many development assistance projects in African countries. Donor agencies complain that insufficient physical infrastructure and technical expertise at the local level generate high transaction costs and, thus, inefficiency in project implementation. *Distribution and institutional channels in Mozambique are weak and thus the US government takes a hegemonic approach* (GFCountryStructure2). The managerial needs of aid administration and implementation are often overlooked. This leads, among others, to slow delivery of assistance and reporting problems [56–59].

Based on the interviews in Mozambique, it is difficult to know if the intention of the Global Fund’s PBF has been communicated on the ground, or if it’s been overshadowed by the administrative burden placed on the principle recipient. A Geneva-based interviewee made a comment that implies, to date, headquarters has recognized the need for change. *[We need to] push down the performance-based funding so that it’s not just done in a committee room in Geneva, but there are these program reviews that are done within countries, … we need to invest much more that there’s a process in-country, and performance-based at the country level* (GFSecretariat2). Until countries take full ownership of this process and are empowered by the intended purpose of performance-based financing, PBF will likely continue to be perceived as an administrative burden and identified as a siloed donor demand.

### Country oversight

The Global Fund does not have country offices. Instead they rely on country teams that are based in Geneva, travel to the country, and are led by a Fund Portfolio Manager with support from 3rd party Local Fund Agents at country level. The team is comprised of programme officers, and legal, procurement, finance, and M&E staff [60]. Team members are responsible for multiple countries. Previously country teams typically visited Mozambique once annually, but with the changes under the New Funding Model, teams visit countries multiple times per year (Multilateral4; OECDPartner2). This increase in frequency has yielded mixed reviews. Many respondents saw it as an improvement because the country teams are becoming more familiar with the realities on the ground and are available for guidance. One respondent contradicted this feedback. *Countries also complain that country teams come too often. There is not enough time to make progress between visits, they are constantly working for the next visit, and this increases the time stress* (GFSecretariat5). Overall, the major concerns voiced by respondents were that country teams are over-worked and are therefore sometimes unsuccessful as the channel for communication, too much depends on an individual (the Fund Portfolio Manager), and the country teams are out of touch with the realities on the ground.

Aside from technical support for proposal development and grant implementation, country teams act as the primary channel of communication with Global Fund headquarters. *The Global Fund does not put things on their website to communicate widely with stakeholders, including at the country level. They rely on Fund Portfolio Managers and people on the country team* (NGO1). Interviewees in-country and in Geneva expressed concern about the reliance on country teams. *The availability of expertise within the team is country-dependent.* … *Personnel are over-worked and over-extended and as a result it is not uncommon for them to take extended leave. This has caused detrimental gaps in communication* (GFCountryStructure1). The Global Fund has recognized some of the issues associated with the burden placed on country teams and *has begun to bring in technical expertise to help* (GFSecretariat5).

One Board Member emphasized the importance of increasing coordination with other development partners in-country to address shortcomings of the country team. *The Global Fund is currently doing stakeholder mapping at the country level so that the network of partners is clear. For now, at least, country teams are staffed with very bright people that regularly visit the country* (GFBoard5). GFBoard2 suggested that some of these concerns could be addressed with clearer expectations for a Fund Portfolio Manager and more effective coordination among constituents. Essentially, there needs to be changes in the hiring of Fund Portfolio Managers combined with diffused powers in oversight of grant implementation.

### In-country coordination

Coordination among donors is central to the harmonization pillar of the Paris Declaration as one of three principles to avoid duplication [61]. Mozambique has a Health Partners Group that meets monthly and brings together all health sector supporters, including representatives of civil society. In 2008 Mozambique signed an International Health Partnership (IHP+) compact [62]. It is a commitment among partners to harmonize and align their support with nationally defined priorities (to the extent that their procedures allow). A Global Fund Board Member identified IHP+ as *the most important opening that we have right now* (GFBoard3) for increased coordination and collaboration with other development partners.

Many more coordinating bodies have been created in Mozambique as a result of absorption challenges. These include the G19 (a group of bilateral donors who provide sector-wide support and coordinate among themselves), the National AIDS Council (Conselho Nacional Contra o SIDA), NAIMA+ (NGOs coordinating body), etc. As one donor representative put it, [the coordinating bodies] *in Mozambique they are a nickel a dozen! This is due to a very weak civil society. If you get an organogram of the Ministry of Health, you will see so many directors and sub-directors, but not many technicians* (GFCountryStructure2).

This has led to conventional donors and local officials spending exorbitant time on coordination, rather than on implementation issues. Coordination among different ‘market players’ involves notably high transaction costs [58]. Yet, integration into a single organization with unified goals involves either high bureaucracy costs [63] or requires very strong leadership [64].

All respondents who represent the Global Fund agreed that coordination at the country-level is vital for successful implementation. A member of the Secretariat commented on coordination as if it is integral to the nature of the Global Fund’s engagement. *We are a contribution model and impact only really occurs when you’ve got other donors in the national program also contributing* (GFSecretariat2). The picture painted by most country-level respondents was very different. Only one participant suggested that s/he was satisfied with the Global Fund’s influence on country-level coordination. *Programmatically they brought a new approach. They forced partners to coordinate more* (Multilateral2). Decision-makers at the Global Fund did voice the need for improvement. Few specifically mentioned country-level coordinating bodies, but they recognized that the only way to avoid duplication is through coordinating with other actors. *There’s definitely more to be done. Fortunately, it’s moving in the right direction. It’s crazy to think you can do appropriate due diligence of a proposal for funding to the Global Fund if you don’t understand what other people are already funding* (GFBoard2). Overall respondents expressed frustration about the Global Fund’s lack of coordination; this was a near-universal theme at the country-level. This was mirrored by GFCountryStructure1’s reflection that the *main criticism during the rounds-based model- the lack of coordination with in-country partners*.

The Global Fund’s challenges with coordination are not unique among implementing agencies in any field of development nor is Mozambique’s ineffective coordination unique among recipient countries (manuscript in preparation). Rather these challenges are symptomatic of a widespread trend in development assistance in most sectors [65, 66].

### Partnerships

The two largest donors to the Global Fund, the United States and France, both contribute 5 % of their pledges to technical assistance (TA) [3]. The 5 % is channelled through their bilateral development agencies or their respective technical bodies founded to support Global Fund principle recipients- Grant Management Solutions and Initiative 5% [67, 68]. Information, personnel, finance, equipment, and supplies are all forms of TA for which applicants are eligible [69]. The latest published list of providers of technical assistance was in 2004 [69]. At the time there were 170 Global Fund-related technical assistance providers (135 organizations and 35 technically-qualified individuals). At the time of writing, the Global Fund website listed six organizations that offer TA. Aside from requests for technical cooperation on community, rights, and gender issues, the Global Fund encourages direct contact with TA providers [43]. *The consultants hired to advise countries on strategies for their concept note development are paid by the partners. … There is a lot of money flowing for Global Fund engagement that is not accounted for. … These activities are coordinated at the country level, at the Development Partners Group* (GFBoard4).

Interviews in Mozambique revealed that partners do more than provide TA, they also step in to fill gaps. *When there are delays / gaps with the Global Fund, other donors step in for support. The other donors’ responses are not formally decided or premeditated* (OECDPartner6). The US government is the largest donor in the health sector; they provide more development assistance for health than all other donors combined (GFCountryStructure2). They often fill gaps in Global Fund support due to the fact that they are both Mozambique’s and the Global Fund’s largest investor.

*The US government is very invested in Mozambique’s success with the Global Fund … The US government is the most involved of all the Global Fund donors both financially but also in terms of coordination at the project-level. All of Global Fund’s activities on the ground in Mozambique are coordinated with PEPFAR and the President’s Malaria Initiative [PMI]* (OECDPartner6).

In Mozambique the US government even has a Global Fund Liaison on the payroll. This position was *created to increase coordination of Global Fund with PEPFAR and [PMI] The position is pay-rolled by USAID, PEPFAR, or US Centers for Disease Control [CDC] depending on the country* (OECDPartner1). Respondents expressed confidence in the liaison and saw the position as the best window into Global Fund support. One interviewee contrasted it with the intended mechanism of coordination.

*The Global Fund liaison is more effective than the Country Coordinating Mechanism because the members of the [CCM] are not actually paid; if the [CCM] were to become institutionalized, it would result in a parallel system (Multilateral5).*

This degree of support from the US government has been invaluable in Mozambique. They are able to identify gaps early on and provide additional support as needed. *The National HIV/AIDS Acceleration Plan [financed by the Global Fund] … is projected to have a massive gap in commodities procurement, which the US government will ultimately need to fill* (OECDPartner6). But interviewees also shared concerns of when the agenda of the US government and the other donors do not align.

*US Congress sets specific targets … that make it imperative for US development activities to follow their own goals. Otherwise Congress will cut funding. … US funding has far more constraints and accountability rules, so that little of it goes directly to the Mozambican government. PEPFAR has a more efficient implementation machine, but –indeed—perhaps the long-term coordination suffers* (GFCountryStructure2).

This response was independently supported by other interviewees (OECDPartners 2 and 3) who touched upon donor relations in Mozambique and the disagreement among the G19 about expectations to hold.

*US government recognizes that Mozambique has weak systems and provides support for the system so as not to set them up to fail but expects them to be a genuine partner and makes changes based on lessons learned. The Global Fund expects the Ministry of Health to apply for funds and then take a “do-it-yourself” approach to systems strengthening, but this fails because they need support* (OECDPartner2).

Some partners question the boundary between the US government and the Global Fund. *In Mozambique criticizing the Global Fund is criticizing US government assistance* (OECDPartner10). Based on discussions with Board Members in Geneva about partners providing TA on the ground, this concern extends up to the highest levels and is perhaps not unique to Mozambique.

A member of the Secretariat reinforced the prospect that there is enmeshment of Global Fund and US government agendas at the highest level. When asked if Global Fund’s donors are coordinating to maximize their contributions the interviewee only discussed coordination with the US government (USG). *The Global Fund has formed a partnership with PEPFAR to avoid duplication. … The USG focuses on service delivery and community-level interventions, and the Global Fund works at the national level* (GFSecretariat5). It is unclear from GFSecretariat5’s response at what level the US government is supposed to share knowledge, and with whom exactly.

The degree of external support required for the Global Fund’s success has raised a debate on accountability. To whom are the providers of TA held accountable?*… the most difficult part of the Global Fund model is the partnership model. It depends on the support of partners and yet it doesn’t have any say over the partners. … I think we need to figure out what that relationship should be* (GFBoard2).

In Mozambique this blurry line extends to the Ministry of Health. *The US government pays for the Global Fund Unit in the Ministry of Health* (OECDPartner6). It’s in the Department of Planning and Coordination and is responsible for coordinating Global Fund projects. In terms of coordination, *the US government is the only bilateral donor that [the Global Fund Unit] has contact with* (GFCountryStructure4).

A new channel was created to manage Global Fund money in 2010/11 in response to the Global Fund’s withdrawal from the health financing basket. Rather than going directly from the Ministry of Finance to the Ministry of Health, money goes through the Global Fund Unit. The combination of burdensome requirements and weak oversight has resulted in the need for an entire unit within the Ministry of Health. *Mozambique is an example of a country undergoing internal reform of reporting systems to adapt to Global Fund requirements* (GFBoard4). The fact that it is paid for by the US government means that it is accountable to the US government and not the Mozambican government.

The Global Fund’s lack of country presence results in inadequate coordination with in-country partners. It also necessitates support from technical partners, for example, the WHO, to address stringent performance-based funding reporting requirements. Additionally they require buttressing by bilateral donors who provide finance to the Global Fund and the countries in which it operates, for example, the US government (Table 2).

### Ability to reform

Overall, there were mixed reviews on what the New Funding Model has, or will, actually change on the ground, but multiple interviewees referred to the Global Fund’s *ability to reform* as one of the defining characteristics of the organization. It was a prominent, recurring theme in interviews.

“*Global Fund has proved in 10 years to have the ability to renew itself*” (Multilateral4).

“*The Global Fund’s New Funding Model addresses inefficiency concerns, and the recent changes in the Global Fund have shown how international organizations are capable of re-inventing themselves*” (GFCountryStructure2).

“*Overall, the Global Fund is learning from its mistakes. … the Global Fund, completed its reformation in one year. It is a ‘learning organization’; it is navigating through a field of opposing forces, and is highly committed to its mission*” (GFBoard4).

Although many of the reforms have not yet addressed concerns about the Global Fund’s overall model, interviewees seemed to be looking beyond this initial transformation. They clarified that although their responses were critical of the Global Fund, they admire the Fund’s work (GFBoard4 and GFBoard5). This sentiment is linked to the organization’s ability to reform- respondents can use opportunities such as interviews to share lessons learned about perceived strengths and weaknesses of the Global Fund with the hope that they are included in the next wave of changes.

“*I think there is commitment from the Secretariat as well certainly from our constituency and others to improve the Funding Model. As Mark Dybul is the first to say, ‘we haven’t got it all right’, this will be an iterative process to continuously improve how the new funding model works*” (GFBoard2).

As one respondent pointed out, the continued success of the Global Fund in Mozambique is important because *the Global Fund is a means of getting around the government; the government does not necessarily reflect societal demands* (OECDPartner3).

## Conclusions

In Mozambique the Global Fund is viewed as an institution that is uniquely capable of reform. Despite the changes with application processes that are associated with the New Funding Model, respondents in both Geneva and Maputo firmly believed that challenges remain in the inherent structure and paradigm of the Global Fund. The lack of a country office has many negative downstream effects including reliance on partners in-country. Due to weak managerial and absorptive capacity, more oversight is required than is afforded by country team visits. In-country partners provide much needed support for Global Fund recipients, but roles, responsibilities, and accountability must be clearly defined for a successful long-term partnership paradigm. Furthermore, decision-makers in Geneva recognize in-country coordination as vital to successful implementation, and other actors in-country would welcome Global Fund engagement. To date, there are no institutional requirements for formalized coordination, and at the time of the interviews the Global Fund has no consistent representation in any in-country coordination groups despite its focus on performance-based finance.

Although the Global Fund’s decision against having local offices in order to encourage local ownership may be justified, the various downstream difficulties suggest that the Global Fund should adopt a more conscientious approach by adapting grant implementation and monitoring procedures to the specific local realities. It should establish procedures that allow room for flexibility while remaining harmonized with headquarter demands. This shifts the onus to headquarters to assess whether what a country reports meets the requirement. The Global Fund could couple these changes with a policy for formalized coordination in-country.

## Additional Portuguese conclusions

Em Moçambique o Fundo Global é visto como uma instituição que é particularmente capaz de se reformar. Apesar das mudanças nos processos de solicitação associados como o Novo Modelo de Financiamento, os consultados em Genebra e Maputo acreditam firmemente que ainda permanecem desafios na estrutura inerente e no paradigma do Fundo Global. A falta de escritórios locais tem muitos efeitos negativos, causando dependência em parceiros nacionais. Devido à capacidade fraca de gerenciamento e de absorção é necessária mais supervisão da que é possível atualmente nas visitas do gerente de carteira em Genebra. Parceiros em Moçambique fornecem apoio aos beneficiários do Fundo, mas as diferentes tarefas, responsabilidades e prestações de contas precisam ser definidas mais claramente para chegar a um paradigma de parceria bem-sucedido a longo prazo. Além disso, os tomadores de decisão em Genebra reconhecem que a coordenação local é necessária para a implementação bem-sucedida; outros parceiros locais apreciariam o envolvimento do Fundo Global. Até agora, não há requisitos institucionais para a coordenação formalizada e, no momento das entrevistas, o Fundo Global não tinha uma representação consistente em nenhum dos grupos de coordenação no país.

Embora a decisão do Fundo Global de não ter escritórios locais possa ser justificada, as dificuldades locais sugerem que o Fundo Global deve adoptar uma abordagem mais consciente, adaptando os mecanismos de subvenção e os procedimentos de monitoramento às realidades locais. Deve-se estabelecer regras que sejam flexiveis e simultâneamente respeitem as demandas da sede central. Isso desloca para a sede central a responsabilidade de avaliar se os relatórios dos países cumprem as exigências. O Fundo Global poderia associar essas mudanças com uma política mais formalizada de coordenação no país.

### Take-home messages

- The lack of a country office has many negative downstream effects including over-reliance on partners in-country.
- Although partnerships provide much needed support for Global Fund recipients, roles, responsibilities, and accountability must be clearly defined for a successful long-term partnership.
- The Global Fund emphasizes coordination at the higher levels of the organization, but country teams’ engagement with other actors in-country is dependent on the Fund Portfolio Manager.
- The Global Fund’s ability to reform is seen as unique, and respondents see its approach as continually evolving.

### Mensagens principais

- A ausência de um escritório no pais tem causado muitos efeitos negativos, incluindo a dependência do Fundo em parceiros locais.
- As parcerias acomodam o apoio necessário aos beneficiários do Fundo Global, mas as tarefas, as responsabilidades e as prestações de contas devem ser definidas mais claramente para ter uma parceria bem-sucedida a longo prazo.
- Fundo Global enfatiza coordenação nos seus níveis mais altos, mas o nível de envolvimento das equipes dentro de Moçambique com parceiros dentro do país depende do gerente de carteira do Fundo.
- A capacidade de reforma do Fundo Global pode ser vista como uma oportunidade única, e os entrevistados consideram sua abordagem como uma evolução continua.

## Additional file

Additional file 1: Semi-structured interview guide. (DOCX 20 kb)

## Abbreviations

CCM: Country Coordinating Mechanism
CDC: US Centers for Disease Control
HSS: Health systems strengthening
IHP: + International Health Partnership
LFA: Local Fund Agent
M&E: Monitoring and evaluation
MDG: Millennium Development Goal
MoU: Memorandum of Understanding
NFM: New Funding Model
NGO: Non-governmental organization
OECD: Organization for Economic Cooperation and Development
OIG: Office of the Inspector General
PBF: Performance-based finance
PEPFAR: the US President’s Emergency Plan for AIDS Relief
PMI: the US President’s Malaria Initiative
PR: Principle Recipient
TA: Technical assistance
TRP: Technical Review Panel
USAID: US Agency for International Development

## Footnotes

1 These include the next eleven emerging economies, N-11 (Bangladesh, Egypt, Indonesia, Iran, Mexico, Nigeria, Pakistan, the Philippines, Turkey, South Korea and Vietnam). Jakovljevic M, et al. BRICS VS. N-11. In: Health Economics and Policy Challenges in Global Emerging Markets. Jakovljevic M, editor. New York: Nova Science Publishers, Inc.; 2016: 1–18. ISBN 978–1–63,484-720-9 (eBook).
