# Supplementary material for: The Global Fund’s paradigm of oversight, monitoring, and results in Mozambique
Source: Global Health. 2017 Dec 12;13:89. doi: 10.1186/s12992-017-0308-7 (PMC5728058; doi:10.1186/s12992-017-0308-7)
Supplement: Additional file 1: — Semi-structured interview guide. (DOCX 20 kb) [file 12992_2017_308_MOESM1_ESM.docx]

*Additional file 1. Semi-structured interview guide*

**Introduction**

“As you may know, our research consortium aims to understand the influence of the current and future trends of non-traditional development assistance for health, especially the increased funding from private foundations, sector or individuals, and other innovative financing schemes on global health architecture, in terms of governance, partnerships, operation and performance.

We are fully aware of the complexity of the topic. The goal of this interview is to obtain the insights of the leaders of selected international health organizations on this topic. We are here to understand the topic, not in any way intending to evaluate, judge, compare or rank the governance or performance of any particular international health organization.

We will maintain your anonymity or acknowledge your contribution to the project, per your wishes. We therefore would highly appreciate it if you could share your thoughts and opinions in regards to our questions without reservation.

The following questions are organized in five sections. i.e. financing, governance, partnership, operation, and outlook of your organization”

**Interviewee Information:**

**Section One: Financing**

**1.1 In your point of view, what have been the major shifts in your organization's financing (e.g. funding sources, scale, areas of funding allocations) over the last 5 to 10 years?**

1.2 What are the underlying reasons for these shifts in financing?

**1.3 What are currently the major challenges and opportunities in terms of financing?**

1.4 In the case when the organization has innovative financing schemes (i.e. financing schemes that tap into new funding sources e.g, airline levies, product-related donations), structure cash flows (e.g. bonds issued against future aid commitments), or channel traditional funding source with new mechanisms), then ask:

**We understand that your organization has been applying innovative financing schemes, such as ….., we wonder what has been the major influence of these innovative financing schemes on the following aspects of your organization, such as financial portfolio and financial sustainability, governance, operation, and performance?** Private FHA Questionnaire **2** of **4**

1.5 What are your organization's plans (if any) to apply innovative financing schemes in the future?

1.6 Do you think innovative financing can be a major source of funding for your organization in the next 5-10 years?

**1.7 What do you think the scale and the structure of your organization's funding scale and sources would change in the next 5-10 years?**

**Section Two: Governance**

**2.1 How has the way that your organization is financed shaped the governance of your organization?**

**2.2 What are the major advantages and challenges of the current governance structure of your organization (e.g. in terms of efficiency in achieving its mission through sound fund allocation and management strategies)?**

**Section Three: Partnerships**

3.1 We understand that your organization has worked closely with many different types of partners. What are the key characteristics you would describe the partnerships that your organization has?

3.2 How has the way that your organization is financed influenced on those partnerships?

**3.3 What are the major advantages and concerns/risks of these partnerships?**

3.4 What are the key lessons to be derived from addressing the concerns (or risks) of these partnerships?

3.5 What could be improved, if any, to enhance collaboration between your organization and other international health organizations and initiatives (e.g. in terms of consultation, resource sharing, joint decision-making or service delivery)?

3.6 What could be improved, if any, to enhance collaboration between your organization and national health institutions?

**Section Four: Operation**

4.1 In what ways does the current financing model of your organization affect its operational strategy and programmes?

**4.2 To what extent has the operation of your organization is influenced by the following entities of your organization: donors, local governments, partners, and beneficiaries?**

| To a great extent | | To some extent | | To a limited extent | | Not at all | |
| --- | --- | --- | --- | --- | --- | --- | --- |
| Donors | ☐ | | ☐ | | ☐ | | ☐ |
| Recipient governments | ☐ | | ☐ | | ☐ | | ☐ |
| Operational partners | ☐ | | ☐ | | ☐ | | ☐ |
| Beneficiaries | ☐ | | ☐ | | ☐ | | ☐ |

**For health ministry officials**

**Overview**

What have been the most influential shifts of external finance for health channelled through your Ministry (in terms of source and magnitude)?

How have these affected resource allocation and the budget for health at the national level?

Were the increases in assistance for health added to the budget or did the funds acts as a substitute? If yes, in which area?

Have changes in development assistance for health led to an increase in interaction and coordination between the Ministries of Health, Finance, and Foreign Affairs in terms of communication and/or resource flows? If yes, please describe. (Follow-up: who initiates such coordination?

Is the coordination formalized? What are the mechanisms? Results?)

**BRICS bilateral**

As defined by the G77’s Ministries of Foreign Affairs meeting in 2009, South-South Cooperation includes five key elements. Could you comment on how your Ministry experiences BRICS partners in terms of the following:

- Partnership;
- Avoiding policy conditionality in governance, economic policy, or institutional reform;
- Structuring assistance to compliment foreign direct investment;
- Emphasizing individual project feasibility rather than long-term debt sustainability;
- Applying domestic development lessons?

Many of the BRICS countries emphasize technical cooperation rather than financial assistance; how do you experience this within your Ministry? Example?

Is this a valued approach?

What are the key influences of this finance channel on national health governance?

**Private finance**

Could you comment on how your Ministry experiences private and/or philanthropic partners for health in terms of the following:

Partnership;

- Avoiding policy conditionality in governance, economic policy, or institutional reform;
- Structuring assistance to compliment direct investment;
- Emphasizing individual project feasibility rather than long-term debt sustainability;
- Applying domestic development lessons?

What are the most notable differences in your Ministry’s experience of private and/or philanthropic partners for health when compared to conventional bilateral or multilateral partners?

What are the key influences of this finance channel on national health governance?

**International Organizations**

Could you comment on how your Ministry experiences public and/or private partners- such as the Global Fund, the GAVI Alliance, Medicines for Malaria Venture, and the World Health Organization- in terms of the following:

- Partnership;
- Avoiding policy conditionality in governance, economic policy, or institutional reform;
- Emphasizing individual project feasibility rather than long-term debt sustainability;
- Applying domestic development lessons?

What are the most notable differences in your Ministry’s experience of public and/or private institutions when compared to conventional bilateral or multilateral partners?

What are the key influences of this finance channel on national health governance?

**Concluding**

How does your Ministry record in-kind assistance for health in the mid-term expenditure framework?

Are there structural/institutional changes needed within your Ministry for effective harmonization of these diverse assistance channels?

Are there any other key points or concerns related to our project that you would like to share?
